# Association between adherence to behavioral intervention and capability well-being among parents of autistic children: a cross-sectional study from China

**DOI:** 10.1186/s12888-024-06394-8

**Published:** 2024-12-18

**Authors:** Huanyu Zhang, Shanquan Chen, Jiazhou Yu, Xuejing Niu, Xue Wang, Li Wang

**Affiliations:** 1 Clinical Big Data Research Center, The Seventh Affiliated Hospital, Sun Yat-sen University, Shenzhen, 518107 China; 2 International Centre for Evidence in Disability, London School of Hygiene & Tropical Medicine, London, WC1E 7HT UK; 3 Jockey Club School of Public Health and Primary Care, The Chinese University of Hong Kong, Shatin, Hong Kong SAR China; 4 International Office, Guilin University of Aerospace Technology, Guilin, 541004 China; 5 Education Practice and Leadership, University of North Dakota, Grand Forks, ND 58202 USA; 6 Shenzhen Research Institute, The Chinese University of Hong Kong, Shenzhen, 518000 China; 7 Brain and Mind Institute, The Chinese University of Hong Kong, Shatin, N.T., Hong Kong SAR China

**Keywords:** Autism, Adherence, Behavioral intervention, Capability well-being

## Abstract

**Background:**

High adherence to behavioral interventions among parents of autistic children contribute to improvement in children’s behaviors and parental outcomes. However, most of previous studies focused on the effects of intervention adherence on health-related well-being among parents, which is inadequate to capture the multi-dimensional parental burden of caring for autistic children. The aim of this study is to assess the association between parents’ adherence to behavioral intervention and their capability well-being.

**Methods:**

A cross-sectional study was conducted among caregivers of autistic children aged 1–17 years with a confirmed diagnosis in China. A total of 213 parents of autistic children who were currently receiving behavioral intervention were considered in this study. Parental adherence to behavioral intervention was evaluated by the 5-item General Adherence subscale of the Medical Outcome Study. Capability well-being was assessed using the Investigating Choice Experiments Capability Measures for Adults (ICECAP-A), including the domains of stability, attachment, autonomy, achievement, and enjoyment. The index scores for each domain were computed based on the preference-based value set in the United Kingdom, ranging from 0 to 1. Multivariate linear regression analyses were conducted to assess the relationship between intervention adherence and parental well-being. Intervention adherence as well as the variables with *p* < 0.1 in the univariate analyses were included in multivariate linear regression analyses.

**Results:**

The mean overall score of ICECAP-A was 0.681 (0.205). In the univariate analyses, intervention adherence was positively associated with stability, autonomy, achievement, and enjoyment (*p* < 0.05), while the relationship between intervention adherence and attachment was not significant (*p* = 0.07). After controlling for the confounders in the multivariate linear regression analyses, intervention adherence was positively associated with achievement (β = 0.0004) and enjoyment (β = 0.0004).

**Conclusions:**

The overall capability well-being among parents of autistic children was worse than that of the general population and caregivers of adult patients with chronic diseases. Adherence to behavioral interventions was positively associated with parental well-being in terms of achievement and enjoyment. Health professionals should involve parents in behavioral interventions and encourage them to implement therapeutic strategies on a routine basis. Customized interventions are warranted to improve capability well-being targeting at vulnerable groups.

## Introduction

Autism spectrum disorder (ASD, hereafter termed autism) is a neurodevelopmental condition that affects 1 in every 100 children globally [1]. Autism is characterized by difficulties in social interaction and communication and the presence of restricted and repetitive patterns of behavior, interests, and activities. Autism poses significant impact for both individuals and their families. Existing literature has revealed that parents and caregivers of autistic children are negatively affected in quality of life and psychological wellbeing, leading to adverse health outcomes including stress, depression, and anxiety [2–5]. It is widely documented that behavioral symptoms of autistic children, including both externalizing (e.g., aggression, self-harm, hyperactivity, etc.) and internalizing (e.g., depression, anxiety, social withdrawal, etc.) behaviors [6], are important predictors of parental stress and burden [7–9]. Addressing these challenges is pivotal for fostering a supportive environment and enhancing the overall well-being of both autistic individuals and their families.

To help the core symptoms of autism, behavioral interventions have been established as the mainstay of non-pharmacological treatments, complemented by other interventions such as speech-language therapy and occupational therapy [10]. Behavioral intervention, rooted in the principles and procedures of Applied Behavior Analysis (ABA), draws upon learning theories like operant and classical conditioning to address behavioral symptoms in autistic individuals [11, 12]. Its goal is to enhance socially significant behaviors while reducing those that may hinder learning or social interactions [13]. Research has shown that long-term involvement in behavioral intervention can significantly impact intellectual functioning, adaptive behavior, and language abilities in autistic individuals, particularly in young children [14]. In addition, parent adherence to behavioral intervention, defined as parents receiving guidance from physicians or clinicians to implement therapeutic strategies with fidelity, is a key component to success of behavioral intervention [15, 16]. Research demonstrates that parents actively involved in implementing therapeutic strategies contribute to sustained positive changes and growth in their children’s behaviors [17]. Beyond the direct benefits for children, high adherence to behavioral intervention among parents was found to be associated with lower parent stress and improved parental quality of life [8, 18]. However, the majority of previous studies were conducted in a research setting, while little is known about the extent to which parents adhere to behavioral recommendations in the absence of professional supervision. Notably, a prior research indicated that parents reported using behavioral skills and techniques significantly less at home than when observed in a research setting [19]. In China, several parent-mediated intervention programs for children with autism have been conducted with a measurement of parent adherence to implement therapeutic skills [20]. Despite a relatively high parent adherence reported in these research programs, there is limited information on parent adherence in the absence of direct supervision. Given that Chinses parents’ reluctance to seek formal and professional support [21], it is unclear whether these programs could be further replicated or disseminated in the Chinese societies. Therefore, it is warranted to measure the degree of parents’ adherence to behavioral recommendations on a routine basis without professional supervision.

Well-being is defined as an individual’s emotional response to what their life is like, generally including physical, mental and social aspects of a person’s life [22, 23]. Considering the burden and challenges of caring for autistic children, most of early studies showed that parents of autistic children are negatively impacted in well-being, leading to physical and mental problems as well as social isolation [2, 3, 24]. Nonetheless, as suggested by a growing number of research, some parents adapt to caring for autistic children and in turn, learn and improve their competence in the childcare process [25, 26]. Therefore, taking into account parental well-being in a positive sense, it provides a more comprehensive perspective to view the effectiveness of behavioral recommendations and in turn, better shape the intervention design and implementation. To provide a broader measure of well-being, we utilized the Investigating Choice Experiments Capability Measure for Adults (ICECAP-A) to measure parental well-being from a positive perspective in this study. This general instrument is intended to measure the following five attributes: stability, attachment, autonomy, achievement, and enjoyment, emphasizing an individual’s ability to achieve important functioning [27].

To our best knowledge, no studies have been conducted to assess the impact of intervention adherence on capability well-being of parents caring for autistic children. Therefore, the main objective of this study is to assess the association between parents’ adherence to behavioral intervention and each domain of their capability well-being. The secondary objective is to identify factors at child, parent, and family level associated with each domain of parental capability well-being.

## Methods

### Study design and participants

A cross-sectional study was conducted from March through September, 2023 in China. Using the convenience sampling method, caregivers of autistic children were recruited through three approaches. First, the questionnaire was distributed through public and private autism intervention institutions to their registered members. Second, several physicians with a specialty of rehabilitation in the department of pediatrics in hospitals recommended the questionnaire to eligible participants. Third, recipients of a publicly-funded early intervention program were invited to join this study. Eligible respondents were able to participate in the study by accessing the QR code which directed them to an online survey, including an explanation of the study’s purpose and protocol, a consent form, and a questionnaire. Participants must click the “consent to participate” button before proceeding to the questionnaire. Withdrawal was allowed for participants at any point in the process of filling in the survey, but only responses without missing answers could be submitted. All the participants were de-identified during and after data collection.

Participants meeting the following criteria were considered eligible in this investigation: (1) parents or caregivers of autistic children; (2) parenting an autistic child aged 1–17 years; (3) a confirmed diagnosis of autism. The cases where children had not been officially diagnosed by a qualified clinician or reported a non-autism diagnosis were excluded from this survey. Consequently, a total of 489 parents/caregivers completed the survey, with 385 providing valid responses. A series of questions were employed to select the participants whose children were currently receiving behavioral interventions. The participants were firstly asked whether their children were currently receiving any intervention (yes/no). Those who answered ‘yes’ were further asked to select one or multiple intervention types their children were receiving, including behavioral, speech, social interaction, occupational, medication, and other treatments. This study exclusively included 213 parents of autistic children who were currently receiving behavioral intervention.

This study was approved by the Research Ethics Committee of the Shenzhen Research Institute, The Chinese University of Hong Kong (PJ-202210B). Informed consent was obtained from all the participants involved in this study. The research was conducted in accordance with the Declaration of Helsinki.

### Study measures

#### Adherence to behavioral intervention

Adherence to behavioral intervention was self-reported by parents using the validated Chinese version of the 5-item General Adherence subscale of the Medical Outcome Study [28]. Participants were required to rate on a five Likert scale from strongly disagree to strongly agree, indicating their general adherence to behavioral intervention in the past four weeks. After reversing the scoring of items 1 and 3, the score of each item is transformed linearly to a 0-100 distribution and the total score is calculated as the average of five item scores. A higher score indicates a better adherence. The internal consistency reliability coefficient (Cronbach’s Alpha) of this adherence assessment tool in the study sample was 0.74.

#### Well-being

Parental well-being was assessed by the validated Chinese version of the Investigating Choice Experiments Capability Measures for Adults (ICECAP-A) [29]. ICECAP-A has the following five attributes: stability (being able to feel settled and secure), attachment (being able to have love, friendship, and support), autonomy (being able to be independent), achievement (being able to achieve and progress), and enjoyment (being able to have enjoyment and pleasure). Each of the five attributes includes one question. The participants were asked to rate on the level of 1 to 4 (1 = full capability, 4 = no capability) for each attribute. To compare the capability well-being of parents caring for autistic children with that of other populations, a preference-based measure of the ICECAP-A was employed in this study. Preference-based data are generally generated from measures that comprise multiple domains to describe individuals’ various aspects of their health [30]. These individual-reported values are then converted to an index score using a selected algorithm (sometimes country-specific), which are based on investigating the general public’s preferences. Due to lack of preference-based data on capability well-being in China, we employed a value set based on general population preferences in the United Kingdom to compute ICECAP-A index scores for each domain, ranging from a scale of 0–1 [27]. A higher index score indicates a better capability well-being. The internal consistency with an overall Cronbach’s Alpha coefficient of the ICECAP-A in the study sample was 0.855.

#### Child, parent, and family characteristics

Child characteristics include age, sex, and severity of behavioral symptoms. The Chinese version of Clancy Autism Behavior Scale (CABS) was used to assess the severity of behavioral symptoms in the current study [31]. Parents rated 14 items of the CABS on three frequency levels, including ‘Never (score of 0)’, ‘Occasionally (score of 1)’, and ‘Often (score of 2)’. The total score was computed as the sum of all 14 items, with a higher score indicating a more severe behavioral symptom. The internal consistency reliability coefficient of the CABS, as indicated by Cronbach’s Alpha, was 0.81 in the current study.

Parent and family variables encompass the role of parent (mother vs. father), parent age, marital status (married, not married, divorced/separated, and other), education level (college or less, bachelor degree, and advanced degree), time on caretaking of the child per day (< 3 h, 3–6 h, 7–12 h, and > 12 h), number of children (1 child vs. >1 child), and annual household income (in the unit of 100,00 0RMB). Since there is limited reimbursement by the government for the medical costs of autistic children in China [32], we did not include a variable describing the funding situation of Chinese families raising autistic children in this study.

### Statistical analyses

Descriptive analyses were performed with mean (standard deviation, SD) for continuous variables and number (percentage) for categorical variables. Univariate analyses of the five domains of well-being included Wilcoxon tests for binary variables, Kruskal tests for categorical variables with more than two categories, and the Spearman correlation tests for continuous variables.

To further assess the association between intervention adherence and parental well-being and identify factors associated with parental well-being, we conducted multivariate linear regression analyses on the five domains of well-being. Intervention adherence as well as the variables with *p* < 0.1 in the univariate analyses were included in multivariate linear regression analyses. Each variable included in the final model was additionally tested for the variance inflation factor (VIF) to avoid collinearity. The results showed that there was no value of VIF larger than 5, indicating the absence of collinearity in all the final models. A *p* value < 0.05 was considered to be statistically significant. All the analyses were completed using R version 4.2.2.

## Results

The study sample was comprised of 189 (88.7%) mothers and 24 (11.3%) fathers, with a mean age of 35.4 (6.3). The majority (85.9%) of the respondents were married, and approximately 60.0% of them had a bachelor or advanced degree. For autistic children, 82.6% are males, with a mean age of 5.6 (SD 2.3; range 2 ~ 17). With regard to the severity of their behavioral symptoms, the mean score of CABS was 15.5 (4.9). Nearly half (47.9%) of the families involved in this study have more than one child. Regarding the key variables, the mean score of intervention adherence was 65.6 (SD 17.5; interquartile range 52 ~ 80), while the mean overall score of ICECAP-A was 0.681 (0.205). More detailed information can be found in Table 1.

**Table 1:** Descriptive statistics for child, parent, and family characteristics and key variables

	All (*N* = 213)
*Child characteristics*	
**Child age**	5.6(2.3)
**Child sex**	
Male	176(82.6%)
Female	37(17.4%)
**Severity in behavioral symptoms**	15.5(4.9)
*Parent and family characteristics*	
**Parent role**	
Mother	189(88.7%)
Father	24(11.3%)
**Parent Age**	35.4(6.3)
**Marital status**	
Married	183(85.9%)
Not married	3(1.4%)
Divorced/Separated	22(10.3%)
Other	5(2.3%)
**Education level**	
College or less	84(39.4%)
Bachelor’s degree	90(42.3%)
Advanced degree	39(18.3%)
**Time on caretaking of the child per day**	
< 3 h	43(20.2%)
3–6 h	44(20.7%)
7–12 h	51(23.9%)
> 12 h	75(35.2%)
**Number of children**	
1 child	111(52.1%)
> 1 child	102(47.9%)
**Annual household income in 100**,**000 RMB**	2.86(6.24)
*Key variables*	
**Intervention adherence score**	65.6(17.5)
**ICECAP score**	0.681(0.205)

Continuous variables are presented as mean (standard deviation, SD) and categorical variables as number (percentage)

In the univariate analyses of the five domains of well-being (Table 2), intervention adherence was positively associated with stability, autonomy, achievement, and enjoyment (*p* < 0.05), while the relationship between intervention adherence and attachment was not significant (*p* = 0.07). For the other child, parent, and family variables, severity in behavioral symptoms was negatively associated with stability, attachment, achievement, and enjoyment (*p* < 0.05). Fathers reported higher scores than mothers in autonomy (*p* = 0.04) and achievement (*p* = 0.01). Educational level (*p* < 0.05) and annual household income (*p* < 0.01) were positively associated with all the five domains of well-being.

**Table 2:** Univariate analyses of the five domains of well-being

	Stability		Attachment		Autonomy		Achievement		Enjoyment	
	Value	*p*-value	Value	*p*-value	Value	*p*-value	Value	*p*-value	Value	*p*-value
**Intervention adherence**	0.137	**0.045**	0.124	0.070	0.142	**0.038**	0.161	**0.019**	0.162	**0.018**
**Child age**	-0.108	0.115	-0.125	0.069	0.044	0.520	0.005	0.937	-0.045	0.516
**Child sex**		0.139		0.130		0.592		0.473		0.978
Male	0.139(0.0678)		0.153(0.057)		0.137(0.048)		0.118(0.044)		0.125(0.049)	
Female	0.159(0.0547)		0.170(0.048)		0.141(0.046)		0.124(0.042)		0.128(0.042)	
**Severity in behavioral symptoms**	-0.259	**< 0.001**	-0.181	**0.008**	-0.126	0.067	-0.155	**0.024**	-0.276	**< 0.001**
**Parent role**		0.842		0.343		**0.040**		**0.011**		0.318
Mother	0.142(0.0671)		0.157(0.056)		0.136(0.048)		0.116(0.044)		0.124(0.048)	
Father	0.148(0.0574)		0.148(0.054)		0.153(0.043)		0.139(0.039)		0.134(0.043)	
**Parent age**	-0.119	0.084	-0.118	0.086	-0.094	0.171	-0.040	0.558	-0.098	0.153
**Marital status**		0.086		0.184		0.121		0.609		0.094
Married	0.147(0.0638)		0.159(0.056)		0.140(0.045)		0.120(0.042)		0.128(0.045)	
Not married	0.0633(0.111)		0.127(0.054)		0.082(0.075)		0.090(0.069)		0.074(0.078)	
Divorced/Separated	0.131(0.0772)		0.135(0.060)		0.127(0.057)		0.114(0.056)		0.120(0.058)	
Other	0.101(0.000)		0.170(0.041)		0.113(0.040)		0.104(0.030)		0.086(0.038)	
**Education level**		**0.012**		**0.004**		**0.048**		**< 0.001**		**0.008**
College or less	0.127(0.0673)		0.142(0.055)		0.130(0.050)		0.105(0.044)		0.115(0.051)	
Bachelor’s degree	0.152(0.0658)		0.164(0.059)		0.147(0.042)		0.125(0.042)		0.137(0.041)	
Advanced degree	0.156(0.0576)		0.171(0.047)		0.132(0.049)		0.133(0.040)		0.122(0.048)	
**Time caretaking of the child per day**		0.194		0.114		0.756		0.090		**0.021**
< 3 h	0.152(0.0617)		0.166(0.051)		0.138(0.055)		0.127(0.044)		0.138(0.039)	
3–6 h	0.148(0.0616)		0.160(0.057)		0.139(0.040)		0.117(0.045)		0.127(0.047)	
6–12 h	0.152(0.0637)		0.165(0.049)		0.139(0.050)		0.127(0.041)		0.134(0.041)	
> 12 h	0.128(0.071)		0.143(0.061)		0.135(0.046)		0.109(0.044)		0.111(0.053)	
**Number of children**		0.478		0.868		0.876		0.800		0.825
1 child	0.146(0.0651)		0.156(0.055)		0.137(0.048)		0.118(0.045)		0.125(0.050)	
> 1 child	0.139(0.0671)		0.156(0.057)		0.138(0.047)		0.120(0.043)		0.126(0.045)	
**Annual household income**	0.301	**< 0.001**	0.262	**< 0.001**	0.212	**0.002**	0.275	**< 0.001**	0.265	**< 0.001**

Values are presented as mean (SD) for categorical variables and correlation coefficients for continuous variables. Bold values indicate statistical significance at *p* < 0.05

The results of multivariate linear regression analyses on the five domains of well-being are presented in Table 3. After controlling for other confounders, intervention adherence was positively associated with achievement (β = 0.0004) and enjoyment (β = 0.0004). Severity in behavioral symptoms was negatively associated with stability (β=-0.003), attachment (β=-0.002), autonomy (β=-0.002), and enjoyment (β=-0.002). An older age of parent was significantly related with a worse stability (β=-0.002). Mothers were more likely to feel a worse achievement than fathers (β=-0.024). Compared to parents without higher education, parents with a bachelor degree had a better performance in stability (β = 0.022), attachment (β = 0.021), achievement (β = 0.016), and enjoyment (β = 0.015), while parents with an advanced degree rated a higher score in attachment (β = 0.026), and achievement (β = 0.023). Parents who spent more than 12 h on caretaking of the child per day had a worse score in enjoyment than parents who spent less than 3 h (β=-0.028).

**Table 3:** Multivariate linear regression analyses of the five domains of well-being

	Stability	Attachment	Autonomy	Achievement	Enjoyment
**Treatment adherence**	0.0003(-0.0002,0.0008)	0.0002(-0.0003,0.0006)	0.0002(-0.0001,0.0006)	**0.0004(0.00005**,0.0007)*	**0.0004(0.0001**,0.0008)*
**Child age**	**-**	-0.002(-0.005,0.002)	-	-	-
**Severity in behavioral symptoms**	**-0.003(-0.005**,-0.001)**	**-0.002(-0.004**,-0.001)**	**-0.002(-0.003**,-0.0006)**	-0.001(-0.002,0.0001)	**-0.002(-0.003**,-0.0008)**
**Parent role (= Mother)**	-	-	-0.017(-0.036,0.003)	**-0.024(-0.043**,-0.006)**	**-**
**Parent age**	**-0.002(-0.003**,-0.0002)*	-0.0008(-0.002,0.0005)	-	-	
**Marital status**		-	-	-	
Married	Reference				Reference
Not married	-0.065(-0.137,0.006)				-0.033(-0.083,0.017)
Divorced/Separated	-0.004(-0.032,0.024)				-0.0004(-0.020,0.019)
Other	-0.029(-0.085,0.027)				-0.030(-0.070,0.009)
**Education level**					
College or less	Reference	Reference	Reference	Reference	Reference
Bachelor’s degree	**0.022(0.004**,0.041)*	**0.021(0.004**,0.037)*	0.014(-0.0003,0.028)	**0.016(0.003**,0.029)*	**0.015(0.002**,0.028)*
Advanced degree	0.018(-0.008,0.043)	**0.026(0.004**,0.049)*	-0.003(-0.022,0.016)	**0.023(0.005**,0.041)*	-0.010(-0.029,0.008)
**Time on caretaking of the child per day**	-	-	-		
< 3 h				Reference	Reference
3–6 h				-0.006(-0.024,0.011)	-0.011(-0.029,0.008)
7–12 h				0.008(-0.009,0.026)	-0.007(-0.025,0.012)
> 12 h				-0.008(-0.025,0.009)	**-0.028(-0.045**,-0.010)**
**Annual household income**	0.001(-0.0003,0.003)	0.0001(-0.0012,0.0013)	0.0002(-0.0008,0.001)	0.0003(-0.0006,0.001)	0.001(-0.0003,0.002)
**F statistics**	4.44***	3.54**	3.57**	4.48***	4.84***
**R-squared**	0.165	0.108	0.094	0.166	0.209

Data are presented as coefficient and its 95% confidence interval. Intervention adherence as well as the variables with a *p* < 0.1 in univariate analyses were further included in multivariate linear regression analyses. Bold values indicate statistical significance at *p* < 0.05. “-” indicate that the corresponding variable was not included in the multivariate regression analyses. *p < 0.05, **p < 0.01, ***p < 0.001

## Discussion

This study found that the level of capability well-being among parents of autistic children (mean: 0.681) in China was considerably lower than that of the Chinese general population (mean: 0.848) [29]. Furthermore, the ICECAP-A score of parents in this study was also lower than that reported by a UK sample of informal caregivers (typically family members) providing unpaid care to adult patients with other chronic conditions (mean: 0.75 ~ 0.81) [33, 34]. The results indicated a heavier burden on capability well-being among parents of autistic children in comparison to the general population and informal caregivers of adult patients. This finding can be explained by the challenges and difficulties that these parents encounter. Increased levels of stress, fatigue, and sleep deprivation have been widely documented among parents of autistic children [35, 36], which are likely to compromise their capability well-being. Furthermore, social stigma, increased caregiving responsibilities, and the high financial burden of caring for autistic children may also inhibit their capacity to socialize with others and make positive changes in their environment [35–39].

This study emphasized the association between adherence to behavioral intervention and capability well-being of parents of autistic children. After controlling for the confounders, parents’ adherence to behavioral interventions was positively associated with achievement (being able to achieve and progress) and enjoyment (being able to have enjoyment and pleasure), while it had no significant relationship with stability, attachment, and autonomy. While raising an autistic child can be challenging, a number of benefits in parenting experience, such as increased spirituality and personal growth have also been reported [40, 41]. Specifically, the significant association found in this study is maybe because a high adherence to behavioral intervention empowered parents by recognizing their expertise and contributions [18, 42], and consequently improved their sense of achievement and enjoyment. Based on these findings, health professionals should consider involving parents in behavioral interventions and encourage them to implement therapeutic strategies on a routine basis. Meanwhile, the potential barriers that are related with poor adherence to behavioral interventions among parents should also be identified in future studies. A recent systematic review of qualitative studies on parental experience of parent-mediated intervention for autistic children revealed that some parents felt unconfident about their parenting skills or overwhelmed by the situation [43]. These parent-related factors may lead to difficulties in implementing the intervention or adhering to therapeutic strategies. Therefore, a qualitative interview is warranted to investigate parents’ perceptions on the barriers to adhering to behavioral treatments and how these difficulties may moderate the relationship between parental adherence and their well-being.

In this study, severity in behavioral symptoms of children was negatively associated with the four domains of parental well-being including stability, attachment, autonomy, and enjoyment. Similar results found that severity in behavioral symptoms was associated with lower parental quality of life [37]. Notably, the relationship between child behavioral difficulties and parental wellbeing could be bidirectional. More severe behavioral difficulties in the child can decrease parental well-being, while in turn, worsen the child’s symptoms. Therefore, it would be beneficial for health professionals to assess the needs of both parents and children and provide parental support to improve outcomes for the whole family.

This study also identified socioeconomic factors at parent and family level associated with parental well-being. In this study, mothers were found to be less likely to feel achievement than fathers. The majority of previous studies revealed that mothers of children with disabilities experience lower quality of life and increased psychological burden than fathers [5, 44–46]. The observed differences between mothers and fathers may be due to maternal caregiving responsibilities. Mothers are most commonly reported as the primary caregiver of autistic children and thus suffer from the majority of caregiving burden [47]. Furthermore, an older age of parent was associated with a worse stability. Compared to parents without higher education, parents with a bachelor degree had a better well-being in stability, attachment, achievement, and enjoyment, while parents with an advanced degree had a better well-being in attachment and achievement. This finding is consistent with a prior research showing that a higher educational level was correlated with better quality of life among carers of autistic children [2]. Parents who spent more than 12 h on caretaking of the child per day were less likely to feel enjoyment than parents who spent less than 3 h. This may arise from the negative impact of caregiving demands among parents of autistic children [7, 35]. Previous studies have been conducted to identify correlates of the overall capability well-being measured by the ICECAP-A in general populations, patients with chronic conditions, and informal caregivers of adult patients [33, 48, 49]. However, little is known on the factors associated with the subdomain of capability well-being among parents of autistic children, making it difficult to compare the current findings with those of prior research. More evidence is needed to identify contributing factors to parental well-being so as to customize interventions targeting at vulnerable subgroups in future clinical practice.

Despite the overall significance of the multivariate regression models, it is important to note that the model accounted for very little variance in the subdomain scores of well-being, especially the scores of autonomy and attachment. Future research is warranted to explore other potential factors that may contribute to parental well-being as well as the mechanism on how parent adherence could affect their capability well-being. For example, a previous study found that treatment burden (i.e., money, time, and energy required to participate in treatment) emerged as a moderator for the association between treatment adherence and parent stress [8]. In addition, existing literature revealed that parents’ belief in the treatment relevance and effectiveness as well as parent self-efficacy could affect parent adherence to behavioral interventions for autistic children [8, 19, 50]. These factors can be further studied to help us better understand parental adherence to behavioral interventions in predicting well-being.

This study firstly explored the relationship between adherence to behavioral intervention and capability well-being among parents of autistic children. The research findings facilitate a more comprehensive understanding of the spillover effect of parental intervention adherence on their well-being. Notably, the capability approach utilized in this study considers a broader dimension of life aspects rather than focusing only on health status, providing a deeper insight into the experience of parenting an autistic child. Additionally, this study focused on the sub-domains of well-being and identified their associated factors at child, parent and family level. The results allow for the prediction of vulnerable groups for poor capability well-being, which can facilitate the formulation of tailored measures.

This study also has several limitations. First, due to the cross-sectional study design, the casual link between intervention adherence and parental well-being was inconclusive and needs further exploration. The significant correlation between intervention adherence and parental well-being found in this study could be bidirectional. Future studies with a longitudinal design may provide deeper insights into the change in intervention adherence over time and its causal relationship with parental well-being. Second, recall bias may exist in self-reported data, especially in the measurement of intervention adherence. The 5-item General Adherence subscale of the Medical Outcome Study is a convenient and easily conducted approach, which has been utilized in several previous studies to measure parent adherence to treatments for autistic children [8, 15]. However, this instrument evaluated parents’ subjective perception of their adherence, where recall bias may exist. A more objective measurement of parent adherence is needed to complement the current findings in further research. Third, this study focused on the capability well-being among parents of autistic children who were currently receiving behavioral interventions, the findings of which are not generalizable to the population who are not seeking behavioral interventions. Furthermore, due to the convenience sampling method, the selection of our study sample is likely biased toward the participants who are more actively involved in behavioral interventions. Those who are not included in this investigation could be those who are unlikely to comply with intervention regimens. Hence, it is necessary to define the non-responding population so as to determine the factors that affect the conclusions of this study. Fourth, the participants mainly consist of mothers in this study, resulting in limited generalizability of the current findings to fathers of autistic children. Although some of previous studies on parenting autistic children incorporated fathers in their samples, little attention was paid to the gender differences during analyses and discussion [51, 52]. Future studies are warranted to focus on fathers’ involvement in behavioral treatments for autistic children and its association with their well-being. Fifth, this study assessed parental adherence to behavioral interventions in daily life in absence of professional supervision. Considering the length of the questionnaire and burden on participants, parents were not asked about the specific intervention strategies or the intervention duration and stage that their children were involved. These factors may also influence parents’ adherence and well-being, and thus needs to be resolved in further research. Lastly, this study only explores the relationship between adherence to behavioral interventions and parents’ capability well-being. Future studies are needed to involve other intervention types that autistic children are prescribed and how adherence to these intervention types affect parental well-being.

## Conclusions

The overall capability well-being among parents of autistic children was worse than that of the general population and caregivers of adult patients with chronic diseases. Adherence to behavioral interventions was positively associated with parental well-being in terms of achievement and enjoyment. Health professionals should involve parents in behavioral interventions and encourage them to implement therapeutic strategies on a routine basis. Customized interventions are warranted to improve capability well-being targeting at vulnerable groups including mothers and those who are older, with a lower education level, and spending extremely long time to take care of the child per day.

## Data Availability

All data generated or analyzed during this study are included in this published article.

## Abbreviations

ASD: Autism spectrum disorder
ABA: Applied Behavior Analysis
ICECAP-A: Investigating Choice Experiments Capability Measure for Adults
CABS: Clancy Autism Behavior Scale
